# Supplementary material for: Pesticide Residues Identification by Optical Spectrum in the Time-Sequence of Enzyme Inhibitors Performed on Microfluidic Paper-Based Analytical Devices (µPADs)
Source: Molecules. 2019 Jul 2;24(13):2428. doi: 10.3390/molecules24132428 (PMC6651370; doi:10.3390/molecules24132428)
Supplement: Supplementary file 1 [file molecules-24-02428-s001.pdf]

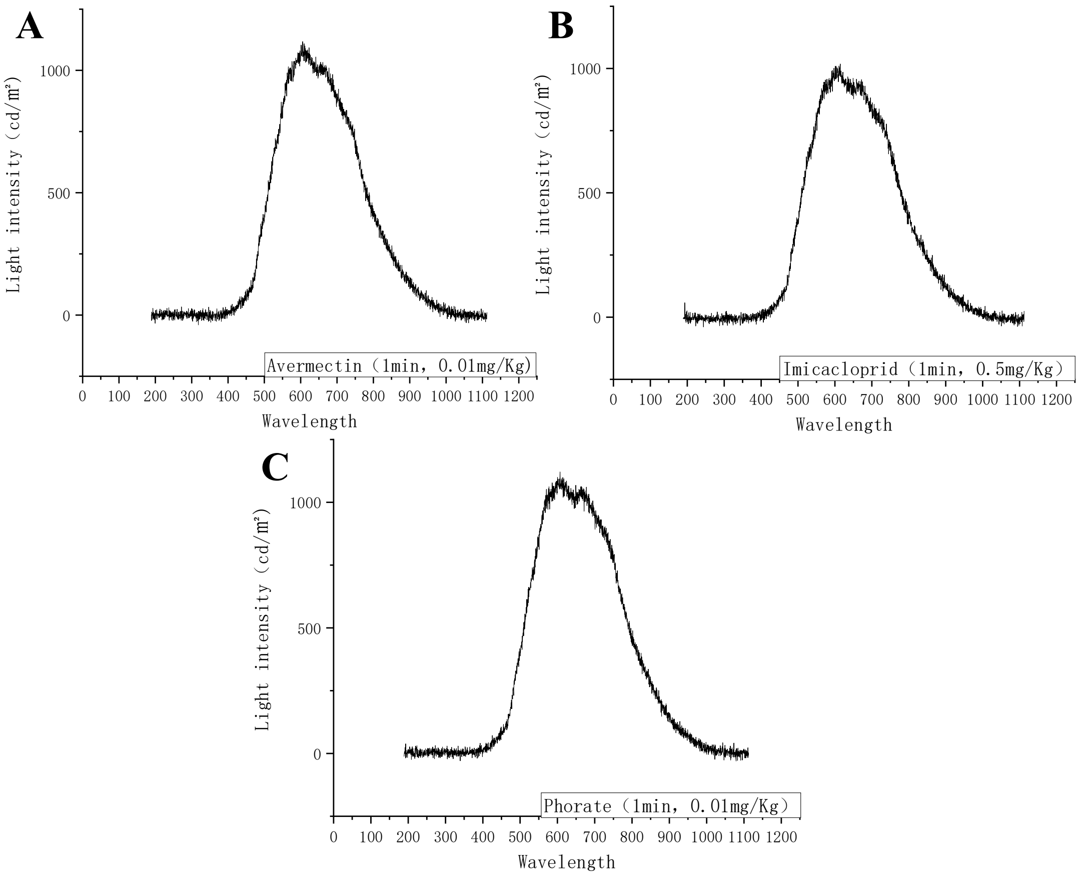

Figure S1. The tested reflected light intensity spectrum from 118nm to 1112nm wavelength, and the figures are recorded by (**A**) avermectin (0.01mg/Kg), (**B**) imidacloprid (0.5mg/Kg), and (**C**) phorate (0.01mg/Kg), when the reaction of the tested chip arrives its 60s.
